# Supplementary material for: Point-of-care testing in a high-income country paediatric emergency department: a qualitative study in Sweden
Source: BMJ Open. 2021 Nov 24;11(11):e054234. doi: 10.1136/bmjopen-2021-054234 (PMC8627407; doi:10.1136/bmjopen-2021-054234)
Supplement: Supplementary data [file bmjopen-2021-054234supp001.pdf]

**SUPPLEMENTARY FILE 1.** Final interview guide used during focus group discussions.

*Experience using current point-of-care technology*

*A. What point-of-care tests do you use today? At the emergency department? In the wards?*
*B. What is your experience with these tests? What aspects do you value? What aspects do you not like?*
    *a. Consider how confident you are in the POCTs: which are the tests you are most confident in using? Least confident? How is reliability? Which POCT provides the most accurate answer and which does not?*
*C. How did you learn to use POCTs? How was the process of learning? Was it easy to learn, and how long did it take? Who taught you how to handle the tests?*
*D. Are there currently available POCTs that you are aware of but do not use? Which? And if so, why?*
*E. Are there any guidelines/protocols for using POCTs? Have they changed over time?*

*What is most important when you use POCTs?*

*A. What are the most important characteristics of a POCT to you?*
    *a. What do you consider the most undesirable characteristics of a POCT?*
*B. Which aspects of POCTs, or concerns about them, makes it less likely for you to use them?*
    *a. Difficult to manage, unpleasant for patients, expensive, or other…?*
*C. In your experience, are there any concerns in health workers when using POCTs? Which?*
    *a. Are there any POCTs you prefer to take twice? Or complement with other examinations?*
    *b. PROBE: What defines "unnecessary sampling"? How do one know it is unnecessary?*
*D. Which are the most important clinical decisions that POCTs help you make?*
*E. In which situations in clinical practice do you believe POCTs are most helpful?*
    *a. If there was no POCTs, what difference would it make at the clinic?*
    *b. How does having POCTs available affect your other duties/your workload?*
    *c. PROBE (for senior doctors): Does the need of POCTs differ when managing a patient directly in contrast to supervising a junior doctor at a distance (e.g., over the phone)?*
*F. How effective and useful do you think your colleagues believe the POCTs are?*
*G. Are there differences among your colleagues in what they think about POCTs?*
    *a. If you compare junior doctors to seniors – have you noticed differences?*
    *b. Or within different specialties?*
    *c. Emergency department compared to wards?*
*H. For whom are POCTs performed mostly? (For diagnostics, for the patient, for the guardians, for the doctor, or for the senior colleague?)*
*I. Who can order a POCT, carry out the test, communicate result to care seeker? What is your opinion of this division of labour?*
*J. Communicating with the patient/care seeker – does the use of POCTs affect your relationship? Do care seekers ask about your choice of tests? Ask for it?*

a. *Do you explain about what you do to the care seeker?*
b. *Do POCTs affect the amount of time you have with your patients?*
c. *Which examinations are replacing the POCTs? Who would perform them?*
d. *POCTs as pedagogical tools, who uses them?*

*The ideal POCT*

A. *How would an ideal POCT improve clinical practice?*
B. *Which conditions or diseases would you like an ideal POCT to diagnose?*
C. *Which characteristics would be desirable in ideal POCTs?*

*Any other questions?*
