# Supplementary material for: Point-of-care testing in a high-income country paediatric emergency department: a qualitative study in Sweden
Source: BMJ Open. 2021 Nov 24;11(11):e054234. doi: 10.1136/bmjopen-2021-054234 (PMC8627407; doi:10.1136/bmjopen-2021-054234)
Supplement: Supplementary data [file bmjopen-2021-054234supp002.pdf]

SUPPLEMENTARY FILE 2. POCTs identified as currently used or known by participants, and requests for future POCTs.

POCTs mentioned in at least one FGD as available to participants and as known from other Swedish clinical settings.		*Requests for specific POCTs and their characteristics, when participants were asked to be visionary*	
**POCTs available to participants**	**POCTs known from other Swedish clinics**	**Specific *ideal* POCTs requested**	**Desired features of *ideal* POCTs**
C-reactive protein (CRP)	Leucocytes	Scanner of airways	Multiplex
Blood gas	Chlamydia trachomatis	Point-of-care ultrasonography	Automated analyses
Urinalysis	Urine drug screening	Appendicitis POCT	Low-cost
mariPOC® Respi test	Full blood count	Cancer POCT	Non-invasive
Respiratory Syncytial Virus (RSV)	Wider blood gas panel	Migraine POCT	Quick
Pregnancy test	Haemoglobin	Procalcitonin POCT	Highly accurate
Glucometer	Erythrocyte sedimentation rate	Blood POCT for meningitis	Fool-proof
Influenza	Transcutaneous carbon dioxide	Rapid urine toxicology POCT	Comfortable for patients
Urine drug screening		POCT for creatinine	Should direct towards correct management
Streptococcus group A		Rapid PCR POCT for urinary tract infections (UTI)	Should quicken diagnostics
Haemoglobin in faeces		Infection aetiology POCT	Test results should be automatically entered into electronic medical record of patients
Blood ketones		Skeletal fluoroscopy for fracture detection	A treatment-guiding POCT
Malaria		Transcutaneous UTI scanner of urine bladder	
Mononucleosis spot			
Transcutaneous bilirubinometer			
Haemoglobin in blood			
Uricult test			
Saturation meter			
